# A Case Study of Argyria of the Nails Secondary to Colloidal Silver Ingestion

**DOI:** 10.7759/cureus.30818

**Published:** 2022-10-28

**Authors:** Kristin Slater, Evelyn Sommariva, Francisca Kartono

**Affiliations:** 1 Dermatology, Lincoln Memorial University DeBusk College of Osteopathic Medicine, Harrogate, USA; 2 Dermatology, Dermatology Specialists of Canton, Canton, USA; 3 Dermatology, MI Skin Center Dermatology Clinic, Northville, USA

**Keywords:** silver supplement, azure lunula, silver, supplement, nail discoloration, colloidal silver, argyria

## Abstract

This case report documents the rare finding of argyria limited to the nails secondary to colloidal silver ingestion. We highlight the significance of early detection of argyria secondary to colloidal silver ingestion and offer photos of the subtle changes in the nails that indicate the development of argyria. With the popularity of over-the-counter supplementation, it is important for medical providers to be aware of early signs of argyria, prior to progressive, permanent pigmentary changes.

## Introduction

Silver has been utilized throughout history for many purposes. In medicine, it has largely been patented and used for its antimicrobial effects [1]. Modernly, silver has been marketed as a health supplement with a range of potential uses [2], although there has not been enough research to establish a well-defined safety profile for over-the-counter supplemental use. Silver ingestion can cause complications including, but not limited to, the risk of developing argyria, a pigmentary condition in which silver particles deposit in the skin, creating blue discoloration [3,4]. Although rare, there are case reports documenting argyria secondary to the ingestion of supplements containing silver [5,6]. In this instance, our patient presented with an early case of argyria secondary to colloidal silver ingestion. The pigmentary change was isolated to the nails and detected through a routine skin examination.

## Case presentation

**Figure 1:**
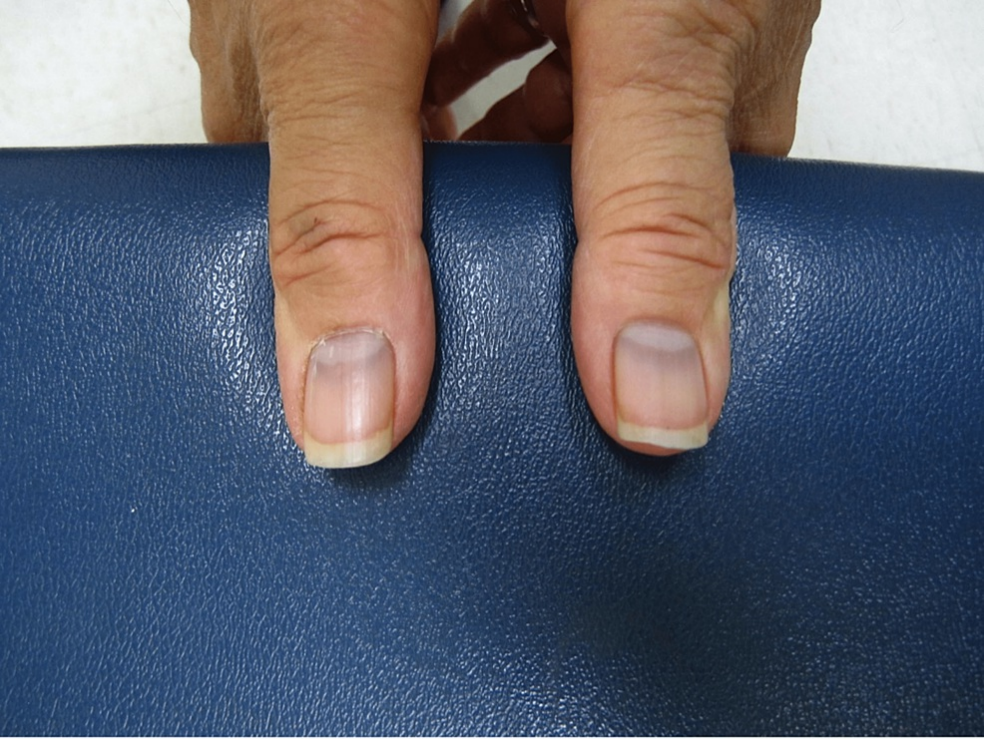
Subtle bilateral blue-gray pigmentary changes in the base of the nails of the first digits.

**Figure 2:**
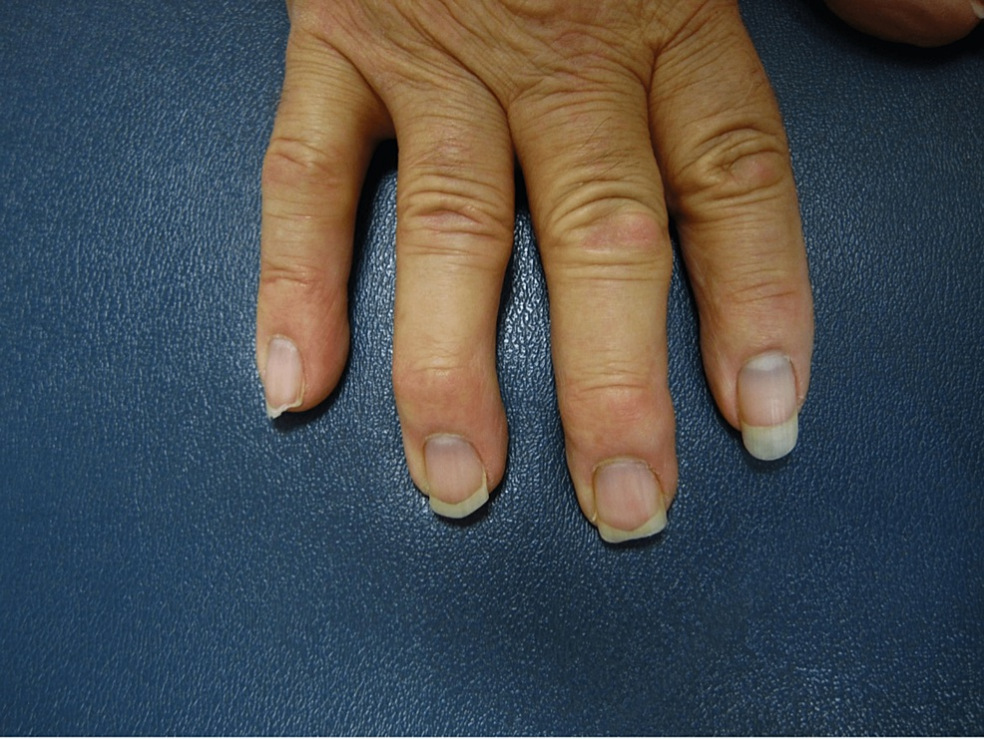
Subtle blue-gray pigmentary changes in the base of the nails of the right hand.

A 79-year-old male presented to the clinic for a routine skin examination. The patient’s previous medical diagnoses included anxiety, arthritis, benign prostatic hyperplasia, a cerebrovascular accident, hypertension, Barrett’s esophagus, and prosthetic arthroplasty of the bilateral hips. During his examination, a mild slate blue-gray hyperpigmentation of the bilateral fingernails was detected (Figure 1 and Figure 2). The patient did not have any remarkable pigmentary changes of the skin. Prior to the nail changes, the patient had started ingesting colloidal silver 30 ppm mixed with liquid silver as a supplement to alleviate symptoms of Barrett’s esophagus. The timing and presentation of the nail changes suggested that the patient had developed early argyria limited to the nails secondary to silver consumption. The diagnosis of argyria localized to the nails (also referred to as azure lunula [4]) was made. The patient was counseled on argyria, including the permanent and progressive nature of the pigmentary changes associated with the condition if silver ingestion continued [4]. To avoid its spread and the progressive discoloration of the nails, the patient was directed to discontinue colloidal silver and liquid silver supplementation. His treatment regimen consisted of the cessation of silver supplementation and the observation of the nails as there are no established treatments for argyria localized to the nails [4]. The expectation was that the progression of the argyria would cease, and the pigmentary changes in the nails would remain stable. Upon follow-up six months later, the condition remained stable, showing no further progression of discoloration.

## Discussion

Although silver has been used in medicine in different forms, its efficacy, risk versus benefit, and safety profile have not been well defined in the context of over-the-counter supplementation. Argyria of the skin and nails is a side effect associated with ingesting silver products [1-3]. It is important that patients are aware of the potential risks of consuming over-the-counter supplements containing silver for medicinal purposes. Argyria of the nails (azure lunula) is an early sign of the condition [4]. Argyria can generally cause noticeable widespread blue-gray pigmentary involvement of the skin [3-6]. Once argyria becomes noticeable, it can cause negative psychosocial effects for the patient [4]. Normally, there are no systemic complications seen with argyria, despite silver’s ability to settle in different tissue types [4]. Some patients with ocular forms of argyria report changes in night vision and other eye-related symptoms [4]. Transient elevation of liver enzymes can be present, but no permanent changes have been described [4]. Most of the long-term systemic effects reported are hard to substantiate beyond a possible causal relationship [4].

Recently, there has been an effort to treat argyria of the skin through laser therapies such as 1064 nm neodymium-doped yttrium aluminum garnet (Nd:YAG) laser, 755 nm alexandrite, and low-fluence Q-switched Nd:YAG [7]. These laser cases show promising results for argyria of the skin but can be painful [7]. Even with these advancements, there are no effective, established treatments for argyria of the nails [4]. Prevention and early detection are preferred. The nails and other localized areas of pigmentary change offer signs of argyria that can be used for early detection prior to larger, generalized skin involvement [4]. In our case, the presentation afforded a clinical diagnosis to be made. Diagnosis can also be made through histopathological analysis, but to indisputably make an argyria diagnosis, energy-dispersive X-ray spectroscopy would be necessary [4]. Other medications to consider as causative agents of argyria mimicking discoloration include minocycline, chlorpromazine/phenothiazines, amiodarone, antimalarial agents, and clofazimine [4]. Differential diagnoses for argyria can include (but are not limited to) cyanosis/cyanotic heart disease, nevi, melanoma, lead poisoning, chrysiasis, ochronosis, and mercury poisoning [4]. This case report highlights the benefit of early detection of agyria development secondary to silver ingestion, prior to significant, permanent progression of nail discoloration. In our case, the patient’s pigmentary changes were identified early and remained mild.

## Conclusions

Argyria can present early in the nails as subtle blue-gray pigmentary changes. If detected early, extensive and permanent discoloration can be avoided. This case demonstrates early nail findings of argyria secondary to colloidal silver consumption and highlights the importance of counseling on the potential risks of silver ingestion.
